# Preoperative Evaluation through Dermoscopy and Reflectance Confocal Microscopy of the Lateral Excision Margins for Primary Basal Cell Carcinoma

**DOI:** 10.3390/diagnostics11010120

**Published:** 2021-01-14

**Authors:** Mihai Lupu, Vlad Mihai Voiculescu, Ana Caruntu, Tiberiu Tebeica, Constantin Caruntu

**Affiliations:** 1 Dermatology Research Laboratory, “Carol Davila” University of Medicine and Pharmacy, 050474 Bucharest, Romania; lupu.g.mihai@gmail.com; 2 Department of Dermatology, “Elias” University Emergency Hospital, 011461 Bucharest, Romania; 3 Department of Oral and Maxillofacial Surgery, “Carol Davila” Central Military Emergency Hospital, 010825 Bucharest, Romania; ana.caruntu@gmail.com; 4 Faculty of Medicine, “Titu Maiorescu” University, 031593 Bucharest, Romania; 5 Department of Histopathology, “Dr. Leventer Centre”, 011216 Bucharest, Romania; tebeica@gmail.com; 6 Department of Dermatology, “Prof. N.C. Paulescu” National Institute of Diabetes, Nutrition and Metabolic Diseases, 011233 Bucharest, Romania; 7 Department of Physiology, “Carol Davila” University of Medicine and Pharmacy, 050474 Bucharest, Romania

**Keywords:** carcinoma, basal cell, microscopy, confocal, dermoscopy, prospective studies, margins of excision

## Abstract

Complete removal of malignant skin lesions with minimal impact on the aesthetic and functional aspects is the ideal of every dermatologic surgeon. Incomplete surgical excisions and tumor recurrences of basal cell carcinomas (BCC) commonly occur due to the subclinical extension of tumor lateral margins. Presently, the lateral excision margins for BCC cannot be objectively assessed preoperatively, dermoscopy proving to be relatively inefficient in this respect. The question is whether BCC lateral excision margins can be precisely determined preoperatively through the use of complementary non-invasive imaging techniques such as dermoscopy and reflectance confocal microscopy (RCM), thus permitting the complete removal of the lesion in a single stage, estimation of the post-excisional defect, and planning an appropriate reconstruction, especially in medical centers where Mohs micrographic surgery is not available. We present the results of a prospective, histopathologically controlled study designed to determine the feasibility of preoperative, non-invasive, in vivo evaluation of the lateral excision margins for primary basal cell carcinoma, through dermoscopy and RCM.

## 1. Introduction

The main objective of dermatologic surgery is to completely remove malignant skin lesions while having a minimal aesthetic and functional impact.

Basal cell carcinoma (BCC) aggressive histopathological subtypes may exhibit subclinical extension of their lateral margins, accounting for numerous incomplete surgical excisions and recurrences. At the moment, the lateral excision margins for BCC cannot be objectively assessed by the dermatological surgeon, dermoscopy proving to be highly dependent on observer experience and relatively inefficient for this application [1,2].

The question behind this study is whether basal cell carcinoma lateral excision margins can be precisely determined preoperatively through the use of dermoscopy and reflectance confocal microscopy (RCM). This would allow for the complete removal of the lesion in one stage, the estimation of the size of the post-surgical defect, and devising an appropriate reconstruction plan, especially in centers where Mohs micrographic surgery is not available. This approach has been considered before and a methodology is already in place [3]. Considering that a skin marker is not visible during RCM examination and can be removed accidentally during surgery, the authors of previous studies [3] have devised an ingenious method for marking tumor margins that provides the investigator with a stable marker during dermoscopy, RCM, and even histological examination: superficial skin incisions. For this purpose, under topical anesthetic, a surgical blade or even a larger gauge needle is used to make very superficial cuts, overlaying previously made markings. Our study builds on this methodology and accounts for new variables that have not been considered previously. The main objective of this study is to determine the viability of preoperative non-invasive in vivo evaluation, through dermoscopy and reflectance confocal microscopy, of the lateral excision margins for primary basal cell carcinoma.

## 2. Materials and Methods

This histopathologically controlled, prospective study included consecutive patients presenting to the Dermatology Department of the Med-As Medical Center and Dermatology Research Laboratory in Bucharest, between September 2018 and November 2020 and represents a continuation of a study from the doctoral thesis of ML [4]. The study was conducted in accordance with the Declaration of Helsinki, and the design and methodology were approved by the Ethics Committee of the “Carol Davila” University of Medicine and Pharmacy, Bucharest through authorization no. 185/2018. All participants gave written informed consent as part of their investigation and treatment procedures, at the time of their registration. Due to the limited information available from previous studies addressing this issue directly, the optimal sample size could not be calculated but was instead estimated to be at least 20 lesions.

The study included patients over 18 years old, with a clinical suspicion of basal cell carcinoma, which agreed to the study, signed the informed consent form, and had lesions in locations accessible to dermoscopic and confocal imaging.

Patients who presented with comorbidities that would encumber the imaging protocol; residual tumors or recurrences after surgical treatments; tumors previously treated with topical therapy (e.g., imiquimod, ingenol mebutate, 5-Fluorouracil); tumors previously treated with photodynamic therapy or radiotherapy; tumors in the proximity of scars, tattoos, or extending to mucosal surfaces; lesions in anatomical regions inaccessible to dermoscopic or confocal imaging were excluded from the study.

Dermoscopy images were captured using the Heine Delta 20T (Heine Optotechnik, Herrsching, Germany) dermoscope connected to a Nikon D80 (Nikon, Japan) DSLR camera through a Heine patented adaptor (Heine Optotechnik, Herrsching, Germany) and the VivaCam (Caliber ID, Henrietta, NY, USA; MAVIG GmbH, München, Germany). A commercially available reflectance confocal microscope (VivaScope 1500; Caliber ID, Henrietta, NY, USA; MAVIG GmbH, München, Germany) was used to acquire confocal images.

All dermoscopy and confocal imaging and their evaluation were performed by an investigator (ML) with over 9 years of experience with dermoscopy and over 4 years of experience with RCM.

**Figure 1:**
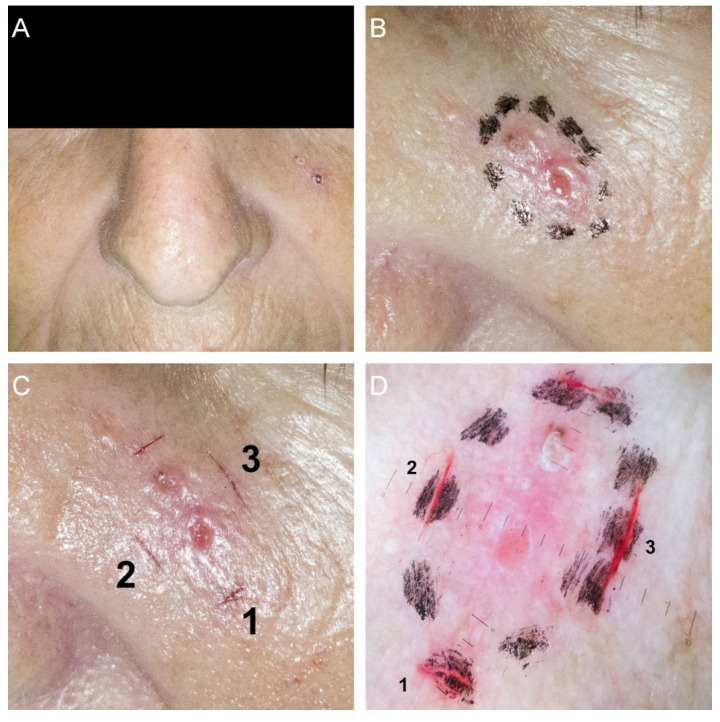
Basal cell carcinoma margin evaluation protocol. (**A**) Clinical overview image; (**B**) Lateral margins drawn after initial dermoscopic examination; (**C**) Superficial incisions overlapping the previously drawn skin markings; (**D**) Dermoscopic image showing the superficial incisions, overlapping skin markings.

The lesion was first assessed clinically. Its location, aspect, and dimensions were noted, followed by the capture of clinical overview images and close-up images (Figure 1A). The lesion and surrounding skin were then cleansed with a 70% ethanol solution. Using a sterile applicator, ROMLA (RAFARM, Athens, Greece) topical anesthetic cream was applied. After 30 min, the anesthetic cream was wiped with sterile gauze and dermoscopic images were captured. The dermoscopic lateral border of the lesion was then drawn 2 mm away from any tumor structure identifiable through clinical examination or dermoscopy (Figure 1B). A new set of clinical and dermoscopic images was then captured. Next, a variable number of small superficial incisions were made using a sterile no. 11 blade, taking care to overlap them on the previously drawn borders. In case of bleeding, hemostasis was readily achieved through compression using sterile gauze and a 3% hydrogen peroxide solution, for 2–3 min. These superficial incisions served a stable marker (approximately 3–4 days) of the lateral lesion margins (Figure 1C,D).

Once hemostasis was obtained, the confocal imaging protocol was initiated. Placing each superficial incision in the middle of the scanning area, a minimum of three 5 × 5 mm mosaics at increasing depths (30 µm increments) were captured, starting at the stratum corneum. The skin on each side of the superficial incision was explored to determine the presence (“positive” RCM margin was defined as the presence of confocal BCC criteria outside the incision or less than 2 mm from the incision on the tumor side) or absence (“negative” RCM margin was defined as the absence of confocal BCC criteria outside the incision or less than 2 mm from the incision on the tumor side) of invasion of the investigated margin. The confocal criteria used to determine the presence or absence of BCC have been described in detail in one of our previously published studies [5], and have been summarized in Table 1.

The imaged lesions were then surgically excised according to their RCM defined lateral margins as follows: “negative” RCM margin lesions were excised along the initial superficial incision, whereas in “positive” RCM margin lesions an extra 3 mm margin was added. To facilitate their orientation in the histopathology lab, all excision specimens were marked with a suture thread at their 12 o’clock position.

After surgical excision, all the specimens were fixed in 10% formalin, vertically sectioned, and stained with hematoxylin-eosin. After histopathological confirmation of the diagnosis of BCC, serial sectioning of the tissue areas containing the superficial incisions was performed. Histopathological examination of all excisional biopsy specimens was done by the same expert dermatopathologist (TT). Tumor presence close (less than 2 mm) or outside the superficial incisions was noted in every case.

The collected data was anonymized, each case receiving a five digit identification number randomly generated through https://random.org, and a secure electronic database containing all the information was created.

### Statistical Analysis

All statistical analyses were performed using the software packages SPSS v20 (IBM, New York, NY, USA) and R [6]. The Chi-square test was used to compare frequencies of categorical variables. The differences in tumor-incision distances measured on dermoscopy, RCM and histopathology were analyzed using Student’s *t*-test, and the relationship between these distances was analyzed through linear regression. The formula used to calculate global accuracy (defined as the overall probability that a lateral margin is correctly classified) was Accuracy = Sensitivity × Prevalence + Specificity × (1 − Prevalence).

If not other specified, data was presented as mean and standard deviation for continuous normally distributed variables, median for continuous non-normally distributed variables, and relative and absolute frequencies for categorical variables. Confidence intervals were set at 95% and a *p* value < 0.05 was considered as statistically significant.

## 3. Results

Eighteen patients (6 males and 12 females) with a mean age of 71.5 ± 6.35 years and a median disease duration of 2 years were included in the study. The youngest patient was 61 years old and the oldest was 82 years old. There were no statistically significant differences between males and females concerning: total number (*p* = 0.157), age (*p* = 0.651) and disease duration (*p* = 0.075).

Clinically, the lesions included in this study were flat (5/20), elevated (13/20), and nodular (2/20). Three lesions were pigmented, three partially pigmented, and fourteen were hypopigmented. Median maximum tumor diameter, measured on dermoscopy, was 11.66 mm (IQR = 6.88), ranging from 4.73 to 25.13 mm.

Most lesions were located in the head and neck region (*N* = 9), followed by the trunk (*N* = 6) and the arms and legs (*N* = 5). Histopathologically, the majority of BCCs were of the nodular subtype (12/20), five (5/20) were superficial, and only three (3/20) had an aggressive histopathological subtype (one micronodular and two infiltrative).

BCC risk evaluation according to NCCN guidelines [7] has revealed four high risk tumors due to their location in the H zone, one high risk tumor in the L zone (due to its size), and three high risk tumors in the M zone (due to their size). Solely concerning histopathological subtype, three out of the 20 tumors were high risk. After cross-classification, half (10/20) of the lesions included in this study were found to be high risk. The median time duration between non-invasive imaging and surgical excision of the lesions was 4.5 days (IQR = 3).

Thirty-two margins in 20 BCCs were explored through RCM. Three margins were destroyed during tissue processing and therefore were not histopathologically examined, leaving 29 margins included in the final analysis. In total, there were four positive and 25 negative RCM margins.

BCC histopathological subtype, anatomical location, clinical characteristics, and tumor-incision distances both in RCM and histology in each case were summarized in Table 2.

A mean number of 1.45 ± 0.51 margins per lesion were evaluated through RCM. The mean examination time was approximately 10 min per margin.

**Figure 2:**
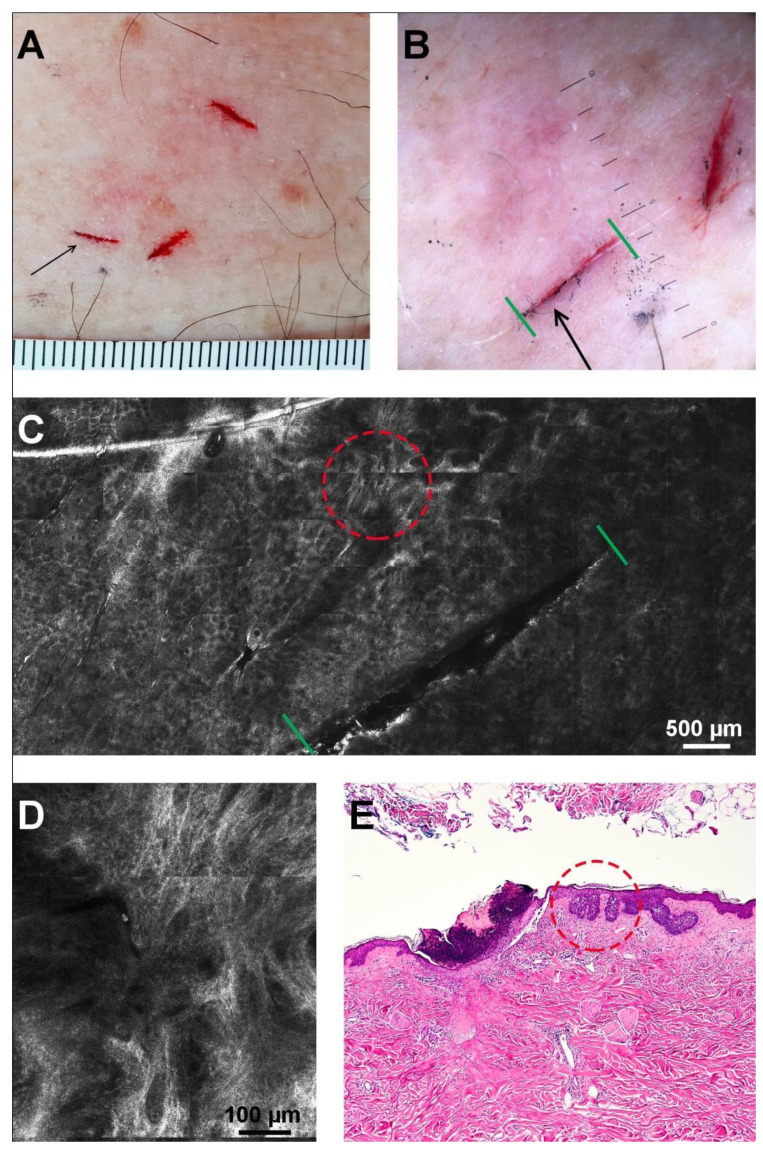
Positive lateral margin in a superficial BCC. (**A**) Clinical aspect with visible superficial incisions (the black arrow indicates the evaluated margin). (**B**) Dermoscopic image of the incision (black arrow; limits are marked by green lines). (**C**) RCM mosaic showing the superficial incision as a dark linear structure (limits are marked by green lines) and BCC foci (red circle). (**D**) RCM detail of the area inside the red circle in panel C, revealing the presence of cords connected to the epidermis. (**E**) Histopathology image showing the superficial incision (black arrow) covered by a crust and cords connected to the epidermis (red circle) in close proximity to the incision (hematoxylin-eosin, original magnification 4×).

RCM examination did not discover BCC criteria extending beyond the superficial incisions in any of the tumors, a finding confirmed by histopathology. RCM showed the presence of BCC confocal criteria within the 2 mm from the superficial incision on the tumor side in four out of the 29 evaluated margins. An example of a “positive” RCM margin has been illustrated in Figure 2. There was agreement with histology in this respect three out of four times, while in one margin (case no. 13) histology showed the tumor was actually further away.

**Figure 3:**
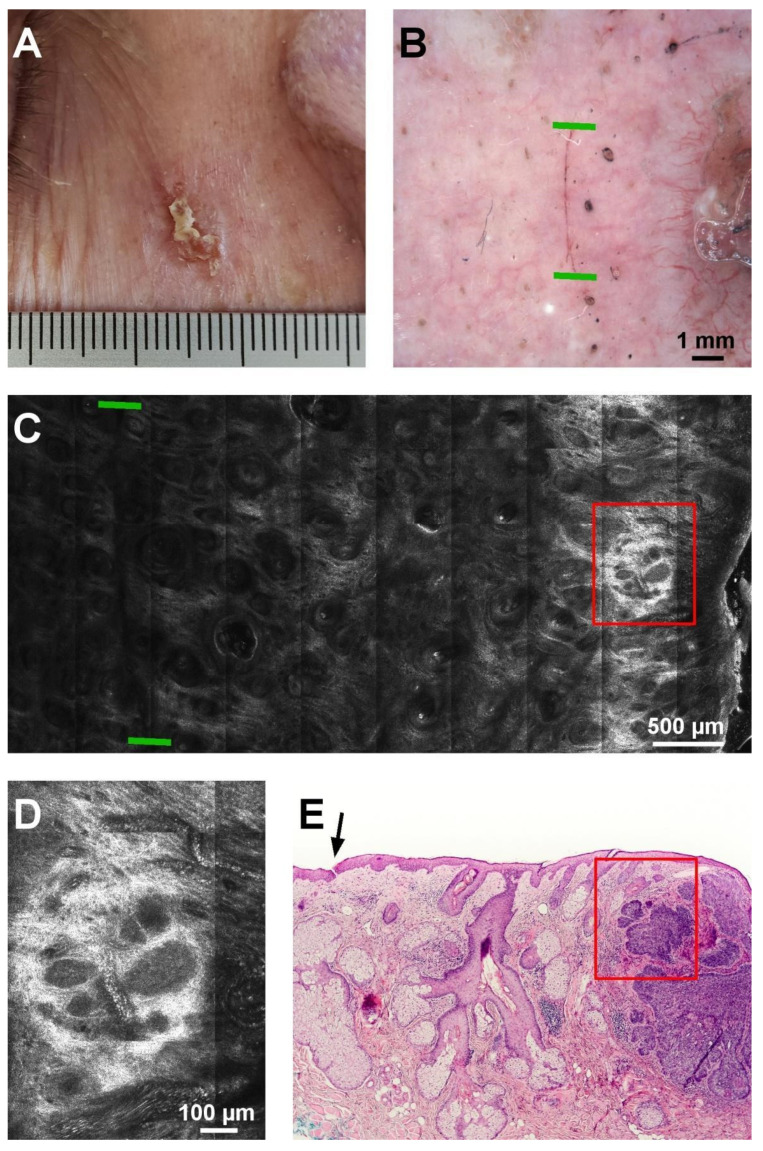
Negative margin in a nodular BCC. (**A**) Clinical aspect of the lesion. (**B**) Dermoscopic image of the incision (black arrow; limits are marked by green lines). (**C**) RCM mosaic showing the superficial incision as a dark linear structure (limits are marked by green lines) and BCC foci (red rectangle). (**D**) RCM detail from the area of the red rectangle in panel C showing tumor islands and hyporefractile silhouettes. (**E**) Histopathology image showing the superficial incision (black arrow) and the tumor mass (red rectangle) at the right of the incision (hematoxylin-eosin, original magnification 4×).

Histopathological examination showed the absence of tumor elements within the 2 mm from the superficial incision on the tumor side in 21 of the 29 evaluated margins, and their presence in eight margins. RCM examination did not discover tumor elements in the 2 mm between the superficial incision and the tumor in 25 of the 29 evaluated margins (Figure 3). These observations have been summarized in Table 3.

Therefore, considering the histopathological examination as the reference standard, RCM sensitivity and specificity for primary BCC lateral margin detection were 0.375 (95% CI 0.08–0.75) and 0.952 (95% CI 0.76–0.99), respectively. The calculated disease prevalence in this study was 0.28 (95% CI 0.13–0.47). The positive and negative likelihood ratios were 7.87 (95% CI 0.95–65.06) and 0.66 (95% CI 0.38–1.13), respectively. The positive and negative predictive values were 0.75 (95% CI 0.26–0.96) and 0.8 (95% CI 0.69–0.87), respectively. The global accuracy of RCM for primary BCC lateral margin detection was 0.79 (95% CI 0.6–0.9).

Tumor-incision distances were overestimated on dermoscopy by 78.98 µm (95% CI −233.644–391.605) compared to RCM (*p* = 0.609) and by 319.606 µm (95% CI 4.99–644.2) compared to histology (*p* = 0.05). Tumor-incision distances on RCM were, on average, 240.626 µm (95% CI 106.39–374.85) longer than those measured in histology (*p* = 0.001). Histological tumor-incision distance exceeded RCM measured distance in only two cases.

The percentage reduction in tumor-incision distances between RCM and histology was, on average, 10.29%. Multiple regression was used to determine if this reduction was influenced by other characteristics such as: gender, age, anatomical location, and number of days from surgery to the release of the histology result. The model was not statistically significant (F(4,10) = 0.705, *p* = 0.606), suggesting that gender, age, anatomical location, and number of days from surgery to histology result had no influence on the percentage reduction in tumor-incision distance between RCM and histology. Furthermore, none of these factors had any individual influence in the percentage reduction between RCM and histology measured tumor-incision distances.

**Figure 4:**
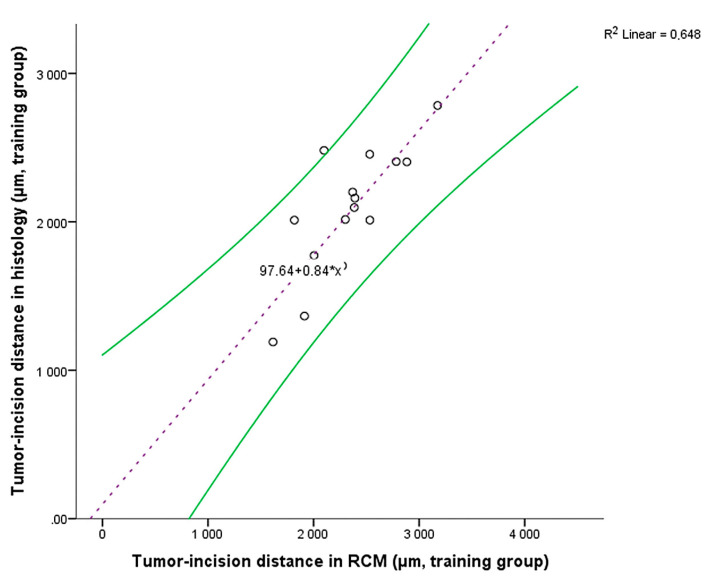
Tumor-incision distances scatter plot in the training group (black circles) and the regression equation (purple dashed line) with 95% confidence intervals (curved green lines).

A linear regression model was used to analyze the relationship between RCM and histopathologically measured tumor-incision distances. Sixteen of these pairs of distances were used in generating the regression equation (training group), which was then used to predict histopathological distances based on RCM measured distances in the test group (13 margins). The linear regression model was statistically significant (F(1,14) = 25.810, *p* < 0.001), with a strong correlation between paired values (R = 0.805). The model explained 64.8% of the variation in our study (R^2^ = 0.648) and 62.3% in the general population (adjusted R^2^ = 0.623). The following regression equation was obtained: Histopathologically measured tumor-incision distance = 97.645 + 0.84 × RCM measured tumor-incision distance (Figure 4).

The mean histopathological tumor-incision distance (measured directly on scale calibrated photomicrographs) in the test group was 2326.75 ± 784.03 µm. The mean tumor-incision distance calculated from the corresponding RCM distances using the regression equation in the test group was 2219.81 ± 520.93 µm. The 106.94 µm difference was not statistically significant (t(12) = 0.829, *p* = 0.423), thus confirming the accuracy of the statistical regression model.

In light of these results, by using the previously determined regression equation, we calculated the corrected histological tumor-incision distances based on the values measured on RCM. The results show that in only six margins were there actually tumor elements less than 2 mm from the incision. We summarized these results in a 2 × 2 contingency table (Table 4).

Therefore, by using the corrected tumor-incision distances, the sensitivity and specificity of RCM for lateral margins of primary BCC detection were 66.67% (95% CI 0.22–0.95) and 100% (95% CI 0.85–1), respectively. The negative likelihood ratio was 0.33 (95% CI 0.11–1.03). The positive predictive value was 100% and the negative predictive value was 92% (95% CI 0.79–0.97). The global accuracy of RCM for lateral margins of primary BCC detection in this scenario was 93.1% (95% CI 0.77–0.99).

## 4. Discussion

Several previous studies, including the ones published by our own group [5,8], have shown the utility of RCM in the non-invasive diagnosis of both benign and malignant skin lesions [9,10,11,12]. Some case reports and case series have demonstrated the potential of RCM for BCC, extra-mammary Paget disease, lentigo maligna [13,14], and amelanotic melanoma preoperative margin evaluation [11,13,14,15,16,17,18,19,20].

Although BCC tumor elements can be precisely identified through RCM, marking of the lateral margins can be a real challenge due to the closed chamber design of the confocal probe head. The metal ring and examination window are fixed to the skin and the scanning head attached to the metal ring is enclosed in a plastic cylinder making it impossible to precisely mark points of interest during RCM scanning of the skin. Moreover, once the metallic ring is removed, the exact position of the areas of interest can no longer be precisely identified. In their attempt to solve this problem, Guitera et al. [17] marked the angles of a small skin area with a skin marker and correlated dermoscopic and confocal images. However, the skin marker is invisible to RCM, making this methodology susceptible to approximation given the possible rotation of dermoscopic images and alignment differences with the RCM scanning head. There have also been attempts at using adhesive paper to delimitate tumor areas [21] for similar purposes, although this method does not provide a histologically identifiable marker. The best approach described so far, and the one we also chose to implement, was proposed by Venturini et al. [3]. The authors marked the tumor margins with superficial incisions before RCM examination, thus creating a stable marker before, during and after RCM examination which could also be used during histopathological examination [22].

However, the authors [3,22] did not explicitly take into account skin biopsy specimen shrinkage [23], and therefore could have under-appreciated the ex-vivo measured tumor-incision distances on histology (although tumor-incision distances were not provided). Specimen shrinkage begins immediately after surgical excision and continues during fixation in formaldehyde (being directly proportional to the time the specimen spends in the fixation agent) and tissue processing for histopathological examination [23,24,25]. Moreover, one study showed that the peritumoral skin contracts more than the tumoral area (mean percentage reduction of 19% vs. 11% for the tumoral area) [23]. Our study takes specimen shrinkage into account and demonstrates, through linear regression models, the reduction of tumor-incision distances between RCM and histopathological examinations. We consider the inclusion of specimen shrinkage in the analysis as one of the strong points of this study. As confirmed by other research [23,24], gender, age, and anatomical location had no influence on the percentage reduction in tumor-incision distances. In our data, the number of days between surgery and the release of the histopathological result did not influence the percentage reduction in tumor-incision distance between RCM and histology. This could mean that, in the cases where there was a longer wait time, other factors were involved, such as histology lab work overload, scheduled equipment maintenance, public holidays, temporary understaffing, etc.

Our results demonstrate that RCM may be employed with high precision in the detection of primary BCC lateral margins (global accuracy of 93.1%). In our sample, dermoscopy under-appreciated the true lateral tumor margins in six of the 29 margins (using RCM only two margins were false-negative), which could have ultimately led to incomplete excisions. On the other hand, dermoscopy is an excellent tried and tested technique for a first-impression diagnosis, overall tumor characterization, and also serves as a guide during RCM imaging. One study corroborates on the possible pitfalls of using dermoscopy to determine the lateral excision margins for BCC [2].

This complementary approach using both dermoscopy and RCM is most useful when confronted with hypopigmented BCC, frequently typified by the lack of specific criteria on dermoscopy [26,27]. Despite this study’s sample size, the superficial incisions could be easily detected clinically, confocally, and histologically in all cases.

One of the important limitations of this approach is the time required for RCM margin assessment. RCM examination was relatively quick, taking approximately 10 min per margin. However, in cases of large tumors with dermoscopically equivocal margins this process must be repeated every 6–8 mm along the perimeter, due to the limited field of view of the generated confocal mosaics [3]. This limitation may be overcome through the use of portable reflectance confocal microscopy devices such as the VivaScope 3000 (Caliber ID, Henrietta, NY, USA; MAVIG GmbH, München, Germany). As previously suggested, the use of a portable confocal device could shorten examination times through the direct evaluation of lesions and margins (incisions) without the need of a metal fixation ring [28] and the possibility of video-mosaicking [29].

Another challenge is excessive bleeding from the incisions, as blood significantly interferes with RCM examination [30]. It is thus optimal that the incisions be kept as superficial as possible and, if bleeding does occur, that rigorous hemostasis is achieved before attempting evaluation through RCM.

We consider the depth at which the tumor-incision distances were measured in histology and RCM as another limitation of the study. Despite our best efforts to match this depth, due to the curving of the tissue during processing we believe this to be a serious challenge, and one which could introduce bias. The steps we took to mitigate this were to firstly note the depth of the block used to measure the distance on RCM and the carefully match this depth on calibrated histological photomicrographs.

A technical limitation that should always be kept in mind is that RCM allows examination of the skin to a depth of about 200–250 µm. Deeper seated lesions cannot be examined with this technique. Hence, the evaluation of the lateral excision margins through RCM is not recommended in the case of infiltrative or micronodular BCCs, due to the possibly deep lateral extension of these histopathological subtypes giving false-negative results. This recommendation is reinforced by the false-negative RCM margin in the case of the micronodular BCC in this study, as well as several observations from previous reports [3]. This depth limitation is overcome by the use of optical coherence tomography (OCT) [31], with the caveat that due to this imaging technique’s resolution the cellular details visible with RCM cannot be assessed. More recently, the development of line-field confocal optical coherence tomography (LC-OCT), an imaging technique with an isotropic resolution of ~1 μm which can produce in vivo images of the skin in B-mode down to a depth of 500 µm aims to mitigate this technical limitation. LT-OCT has been used to image various skin lesions in vivo, including BCC [32]. Very similar to RCM, LT-OCT images have also been found to strongly correlate with conventional histopathological images [32].

Further, larger sample studies are necessary to evaluate the efficiency of this procedure in a surgical setting [18,33,34] regarding BCC, but also other cutaneous malignancies such as squamous cell carcinoma and melanoma.

The use of handheld confocal probes within this margin evaluation protocol can lead to a reduction in RCM examination times. Prospective, randomized studies comparing the efficiency of RCM versus Mohs technique in obtaining a first tumor-free margin, and tumor-recurrence rates between these methods, can also be expected.

## Figures and Tables

**Table 1:** Confocal criteria for the diagnosis of basal cell carcinoma.

Histopathological Subtype	Confocal Criterion
**Nodular BCC**	thick collagen bundles surrounding tumor islands
	increased vascularization
	big tumor islands (diameter > 300 µm)
**Superficial BCC**	cords connected to the epidermis
**Aggressive BCC**	hyporefractile silhouettes

BCC, basal cell carcinoma.

**Table 2:** Basal cell carcinomas (BCC) histological subtype, anatomical location, clinical characteristics, and tumor-incision distances in reflectance confocal microscopy (RCM) and histology.

Case No.	Sex	Age (Years)	Histological Subtype	Tumor Location and Clinical Characteristics	Tumor-Incision Distance, RCM (µm)	Tumor-Incision Distance, Histology (µm)	Tumor-Incision Distance Percentage Reduction between RCM and Histology (%)
1	M	77	nodular	Right zygomatic, 14.4 × 7.42 mm, flat, hypopigmented	1: 2384.993 2: 2783.069	1: 2096.367 2: 2453.325	1: 12.1 2: 11.85
2	F	70	nodular	Scalp, 9.57 × 9.41 mm, elevated, partially pigmented	1: 2099.000 2: 2523.810	1: 2481.481 2: 2240.014	1: −18.22 2: 11.24
3	F	82	nodular	Left pre-auricular, 16.26 × 11.55 mm, nodular, hypopigmented	1: 3174.603 2: 2333.333	1: 2784.000 2: 1272.176	1: 12.3 2: 45.48
4	F	64	nodular	Left zygomatic, 10.59 × 5.71 mm, elevated, partially pigmented	2783.069	2405.983	13.55
5	M	78	superficial	Posterior trunk, 16.64 × 12.79 mm, flat, hypopigmented	1615.544	1190.476	26.31
6	M	74	nodular	Forehead, 8.6 × 7.84 mm, elevated, pigmented	1: 2274.000 2: 2256.624	1: 1703.704 2: 2243.386	1: 25.08 2: 0.59
7	F	80	nodular	Left nazolabial fold, 5.86 × 4 mm, elevated, hypopigmented	1: 2391.534 2: 3301.021	1: 2159.896 2: 3164.021	1: 9.69 2: 4.15
8	F	68	superficial	Posterior trunk, 13.28 × 13.14 mm, flat, hypopigmented	2552.614	2227.513	12.74
9	M	61	nodular	Scalp, 10.65 × 8.2 mm, elevated, pigmented	4074.866	4050.774	0.59
10	F	74	micro-nodular	Right temporal, 11 × 9.59 mm, flat, hypopigmented	2104.061	1851.852	11.99
11	F	72	nodular	Left pre-auricular, 6.95 × 4.61 mm, elevated, partially pigmented	1: 2300.782 2: 2136.734	1: 2015.784 2: 3104.743	1: 12.39 2: −45.3
12	M	66	nodular	Posterior trunk, 6.74 × 6.47 mm, elevated, hypopigmented	2370	2200.321	7.16
13	F	65	nodular	Posterior trunk, 18.53 × 9.06 mm, elevated, hypopigmented	1817.772	2011.811	−10.67
14	F	81	infiltrative	Right arm, 4.73 × 3.48 mm, flat, hypopigmented	2532.065	2455.203	3.04
15	F	65	infiltrative	Righ arm, 5.7 × 4.32 mm, elevated, pigmented	1913.7	1365.012	28.67
16	M	67	superficial	Lateral trunk, 12.33 × 6.24 mm, elevated, hypopigmented	1: 2031 2: 2759.154	1: 1666.900 2: 2349.458	1: 17.93 2: 14.85
17	-	-	superficial	Anterior trunk, 13.78 × 12.03 mm, flat, hypopigmented	2241.728	2100.394	6.3
18	-	-	superficial	Left arm, 10.77 × 10.02 mm, elevated, hypopigmented	1: 2883.071 2: 1604.899	1: 2403.825 2: 1048.448	1: 16.62 2: 34.67
19	F	74	nodular	Left arm, 12.01 × 8.16 mm, nodular, hypopigmented	2533.333	2011.742	20.59
20	F	69	nodular	Right thigh, 25.13 × 15.63, elevated, hypopigmented	1: 2004 2: 2171.188	1: 1773.070 2: 2141.732	1: 11.52 2: 1.36

BCC, basal cell carcinoma; RCM, reflectance confocal microscopy; No., number; M, male; F, female.

**Table 3:** BCC margin positivity on RCM versus histology.

	Histological Positive Margin	Histological Negative Margin	Total
**RCM positive margin**	3	1	4
**RCM negative margin**	5	20	25
**Total**	8	21	29

BCC, basal cell carcinoma; RCM, reflectance confocal microscopy.

**Table 4:** BCC true and false positive and negative margins after histopathological tumor-incision distance correction.

	Histopathologically Positive Margins	Histopathologically Negative Margins	Total
**RCM positive margins**	4	0	4
**RCM negative margins**	2	23	25
**Total**	6	23	29

BCC, basal cell carcinoma; RCM, reflectance confocal microscopy.

## Data Availability

The data presented in this study are available on request from the corresponding author. The data are not publicly available due to privacy reasons.
